# Digital Marine Bioprospecting: Mining New Neurotoxin Drug Candidates from the Transcriptomes of Cold-Water Sea Anemones

**DOI:** 10.3390/md10102265

**Published:** 2012-10-18

**Authors:** Ilona Urbarova, Bård Ove Karlsen, Siri Okkenhaug, Ole Morten Seternes, Steinar D. Johansen, Åse Emblem

**Affiliations:** 1 RNA and Transcriptomics Group, Department of Medical Biology, Faculty of Health Sciences, University of Tromsø, N9037 Tromsø, Norway; Email: ilona.urbarova@uit.no (I.U.); baard.ove.karlsen@northernbiolabs.no (B.O.K.); siri.okkenhaug@gmail.com (S.O.); ase.emblem@uit.no (A.E.); 2 Pharmacology Group, Department of Pharmacy, Faculty of Health Sciences, University of Tromsø, N9037 Tromsø, Norway; Email: ole-morten.seternes@uit.no; 3 Marine Genomics Group, Faculty of Biosciences and Aquaculture, University of Nordland, N8049 Bodø, Norway

**Keywords:** deep sequencing, drug discovery, marine bioprospecting, neurotoxin, sea anemone, transcriptomics

## Abstract

Marine bioprospecting is the search for new marine bioactive compounds and large-scale screening in extracts represents the traditional approach. Here, we report an alternative complementary protocol, called digital marine bioprospecting, based on deep sequencing of transcriptomes. We sequenced the transcriptomes from the adult polyp stage of two cold-water sea anemones, *Bolocera tuediae* and *Hormathia digitata*. We generated approximately 1.1 million quality-filtered sequencing reads by 454 pyrosequencing, which were assembled into approximately 120,000 contigs and 220,000 single reads. Based on annotation and gene ontology analysis we profiled the expressed mRNA transcripts according to known biological processes. As a proof-of-concept we identified polypeptide toxins with a potential blocking activity on sodium and potassium voltage-gated channels from digital transcriptome libraries.

## 1. Introduction

Marine bioprospecting has significant potential for the discovery of novel drugs, nutritional supplements and industrial biotechnology. The traditional approach is to extract bioactive compounds from a sample by bioassay-guided fractions and thereafter determine the structure, chemical composition and exact function [1]. *In silico* analysis and genetic discovery of marine biomolecules complement the traditional methods in bioprospecting [2,3,4]. By sequencing genes, genomes and transcriptomes, the search for gene homologs, motifs or transcripts with a certain expression profile can be identified.

Two years ago we reviewed the concept idea of using massive parallel deep sequencing of transcriptomes in the systematic screening for marine drug candidates [2]. Deep sequencing technologies have revolutionized the field of biology by making it achievable to sequence whole transcriptomes of non-model organisms at a relative low cost [5,6,7]. Three deep sequencing platforms have dominated the research of whole transcriptome analysis; the 454 pyrosequencing platform from Roche, the Genome Analyzer platform from Ilumina Sequencing technologies, and the SOLiD ligation sequencing from Life technologies [8]. These technology platforms produce raw sequence reads with a length from 50 to more than 500 nucleotides, generating billions of nucleotides in a single run. 

The class Anthozoa, where sea anemones and corals belong, has an interesting evolutionary position as one of the most basal eumetazoans, and recent genome analyses have revealed a gene content and structure more similar to vertebrates than earlier expected [9,10]. Corals and sea anemones are mainly sessile, and must adapt to the changing environment, catch prey, and defend themselves from predators and disease-causing agents. Thus, corals and sea anemones are promising candidates in bioprospecting of novel drug compounds [2,11,12]. Understanding coral and sea anemone biology is also essential in preserving the biodiversity that inhabit coral reefs. Despite this, only a limited number of Anthozoa transcriptomes have been sequenced. *Acropora millepora* and *A. palmate*, the main reef-builders of the Great Barrier Reef and the Caribbean reefs, respectively, are both subjected to genome and transcriptome sequencing. 454 transcriptome data from the coral larvae were already previously annotated with names and Gene Ontology (GO) terms [13,14], and applied in comparative and environmental studies. Expressed sequence tag (EST) and transcriptome projects have been initiated for selected sea anemones and corals [15,16] including the upcoming sea anemone model *Aiptasia *[17,18,19]*.* Many EST analyses are however still carried out by the use of Sanger sequencing of cloned libraries [3,20,21].

Neurotoxins are produced by a diverse group of organisms, including sea anemones [22,23]. Typically, they are relatively small peptides with conserved cysteine residues, forming disulfide bridges critical for the peptide structure [24]. Many neurotoxins are translated as inactive precursors with an *N*-terminal leader peptide sequence and a *C*-terminal mature peptide toxin. The active peptide is produced by proteolytic cleavage of a conserved dyad (Lys-Arg) [25]. Neurotoxins block cellular processes in the nervous system and other tissues by binding to voltage-gated ion channels. In sodium channels, six neurotoxin binding sites have so far been identified [26]. These neurotoxins either block the channel pore, or modify the gating, which causes a massive release of neurotransmitters and inactivation delay. The potassium channels represent a diverse group of proteins and a variety of potassium channel toxins block these channels by different mechanisms and thereby facilitate release of the neurotransmitter acetylcholine. Potassium toxins act in synergism with other peptides such as anti-cholineesterases and sodium channel toxins [27].

**Figure 1:**
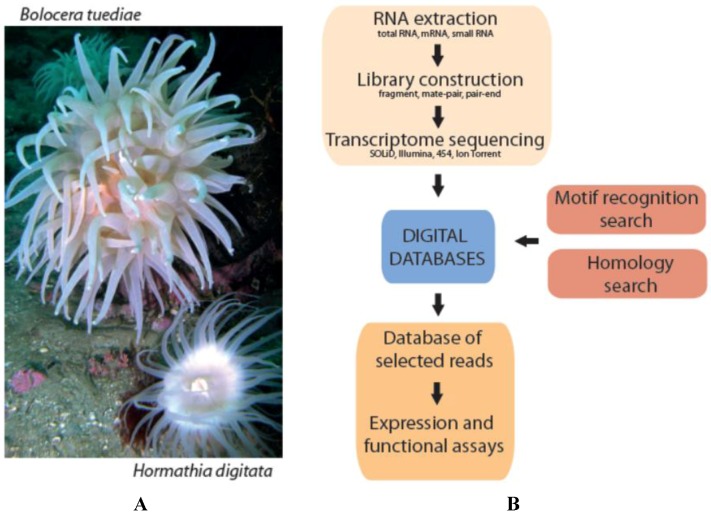
(**A**) The cold-water sea anemone species *B. tuediae* and *H. digitata* included in this study; (**B**) Flowchart describing the pipeline in digital bioprospecting from RNA extraction to prediction of candidate biomolecules, which can be expressed in functional trials. Photo by SDJ.

We used 454 GS FLX Titanium deep sequencing to profile transcriptomes and identify expressed genes and derived gene products in the adult polyp stage of two distantly related cold-water sea anemone species, *Bolocera tuediae* and *Hormathia digitata* (Figure 1A). Here we present a protocol for digital marine bioprospecting in order to identify new peptide drug candidates derived from transcriptome sequencing libraries. 

## 2. Results and Discussion

### 2.1. Transcriptome Sequencing and Assembly

Transcriptome sequencing was performed by 454 GS FLX Titanium (pyrosequencing) and resulted in 546,903 and 546,846 quality-filtered sequencing reads after adapter trimming with an average size of 333 and 331 nt from *B. tuediae* and *H. digitata*, respectively (Table 1). From these reads, 64,442 (*Bolocera*) and 54,293 (*Hormathia*) contigs were assembled. Transcripts found in one copy number (single reads) counted for about 20% of all transcripts. The raw sequence data from *B. tuediae* and *H. digitata* transcriptomes in this study were archived at NCBI’s Sequence Read Archive (SRA) under the accession number SRP011434.

**Table 1:** Transcriptome sequencing and assembly ^a^.

Species	Reads/Contigs	Number	Average size (nt)	Total nt
*B. tuediae*	Raw reads	547,061	547	299,232,484
	Trimmed reads	546,903	333	182,128,133
	All contigs	64,442	591	38,101,858
	Large contigs	5072	1380	6,997,895
	Single reads	118,104	279	33,008,862
*H. digitata*	Raw reads	546,974	543	296,833,666
	Trimmed reads	546,846	331	181,169,361
	All contigs	54,293	613	33,255,104
	Large contigs	5083	1430	7,272,471
	Single reads	105,695	260	27,786,964

^a^ Number of sequencing reads obtained from 454 pyrosequencing of the transcriptomes of the two sea anemones *B. tuediae* and *H. digitata*. Raw Reads, represent all sequence reads obtained from the transcriptome sequencing. Trimmed reads, represent raw reads after trimming of key tag (TCAG) at the 5′ end and removal of low quality and adapter sequences. All contigs, represent all contigs assembled by MWG Eurofins. Large Contigs, represent assembled contigs with size larger than 1000 bases. Single reads, represent reads that are only found in one copy number in the dataset.

### 2.2. Annotation and Gene Ontology Analysis

**Figure 2:**
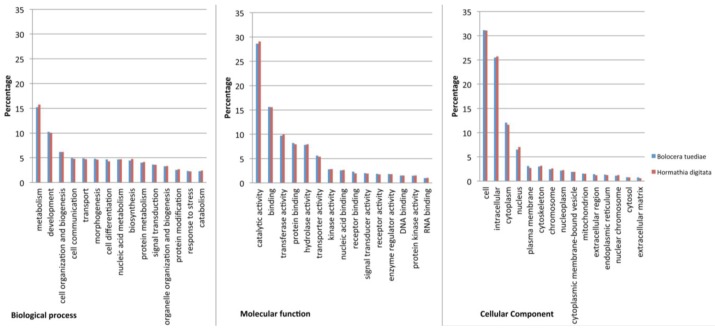
Gene Ontology (GO) assignment for *B. tuediae *and *H. digitata* from 454 pyrosequencing. All assembled contigs together with single reads were blasted and annotated. For the 182,546 and 159,988 contig sequences together with single reads for *B. tuediae *and *H. digitata*, respectively, 104,622 and 128,814 GO terms in total were assigned. Furthermore, 127 GO slim ancestor term were assigned to both species. Transcripts were annotated in three main categories: cellular components, molecular function and biological processes. Top 15 classes from each GO category were chosen as representatives for transcriptome comparison. A single transcript could be assigned in more than one category.

All contigs together with single reads (182,546 sequences for *Bolocera* and 159,988 for *Hormathia*) were analyzed in Blast2GO. From the 64,442 *Bolocera* contigs, 25,447 (40%) had BLAST hits to known proteins. Furthermore, 17,153 (27%) were assigned with GO terms and 11,666 (18%) were annotated. As expected, a much smaller fraction of the 118,104 single reads had BLAST hits (21,268 reads corresponding to 18%). For the 54,293 *Hormathia* contigs, 22,210 (48%) had BLAST hits, 13,787 (25%) were assigned with GO terms, and 9514 (18%) were annotated. From 105,695 single reads for *Hormathia*, 22,864 (22%) had BLAST hits. Additionally, 3674 and 4680 single reads were annotated for *Bolocera* and *Hormathia*, respectively, and assigned GO terms added to the final analyses. The GO terms assigned to the contig sequences were then exported to CateGOrizer, and GO slim analyses were run externally and graphs were produced in Microsoft Excel. GO slim terms are higher-level GO ontology categories, which provide a better profile for specie comparison [28]. The sequences were then classified according to three main GO categories, molecular function, biological process and cellular components and visualized in bar charts (Figure 2). After GO slim was performed, there were 8655 GO terms in total for *B. tuediae*, assignments to the biological process category made up the majority (5,562; 64%), followed by molecular function (2180; 25%) and cellular components (913; 11%). From 9066 GO terms for *H. digitata*, biological process category was also represented in majority (5799; 64%), compared to molecular function (2257; 25%) and cellular components (1010; 11%). *B. tuediae* and *H. digitata* belong to the distantly related families Actiniidae and Hormathiidae, respectively, of the order Actiniaria [29]. Interestingly they possess very similar transcriptome profiles in adult polyps (Figure 2). This may be explained by the identical environmental growth conditions since these sessile individuals were sampled side-by-side at 25 meters depth (Figure 1A). Additionally, both species showed a very high similarity to *Nematostella vectensis* sea anemone [9]. Here, 17,295 out of 25,447 and 18,277 out of 26,210 TOP-BLAST hits for *Bolocera* and *Hormathia*, respectively, were assigned to *N. vectensis*.

### 2.3. A Protocol for Digital Marine Bioprospecting

A main objective of this study was to present a workflow for digital bioprospecting. The reported protocol is based on whole transcriptome sequencing of a desired organism, recognizing sequences or motifs by bioinformatical tools, and thereafter expressing the candidate gene to perform further functional characterizations. A flowchart of the approach is presented in Figure 1B.

At least two different approaches can be pursued. First, regular BLAST homology searches can be performed at different stringencies. Stringencies must be evaluated for each query, depending on expected conservation between query sequence, database, and acceptable degree of false positives. Evaluation can be performed by inspection of reciprocal searches when applying different parameters. Sequencing data are arranged into a local database that represents a digital library, and annotated homologs of desired molecules are then employed as the query, either at the nucleotide or amino acid sequence level. The database can be utilized as a collection of crude sequences from the original reads, or sequence assemblies can be produced, representing longer and more complete contigs. One drawback of databases with assembled contigs is the risk of producing false assemblies due to the presence of more than one gene copy or closely related homologs, as well as alternative splicing or RNA editing. This can however be largely avoided by stringent settings for contig assemblies. False assemblies can be a challenge for toxin peptides, which often are expressed together with closely related isoforms [30]. 

Searches in this study were performed on assembled contigs and on single reads. First, 284 sodium and 268 potassium channel toxins from different species were downloaded from SwissProt/UniProtKB protein database and used as query sequences. After closer examination of possible hits, it was concluded that candidate toxins are more similar to published sea anemone toxins and 78 annotated sea anemone neurotoxins were therefore used as query sequences for follow-up analyses (Table S1).

**Figure 3:**
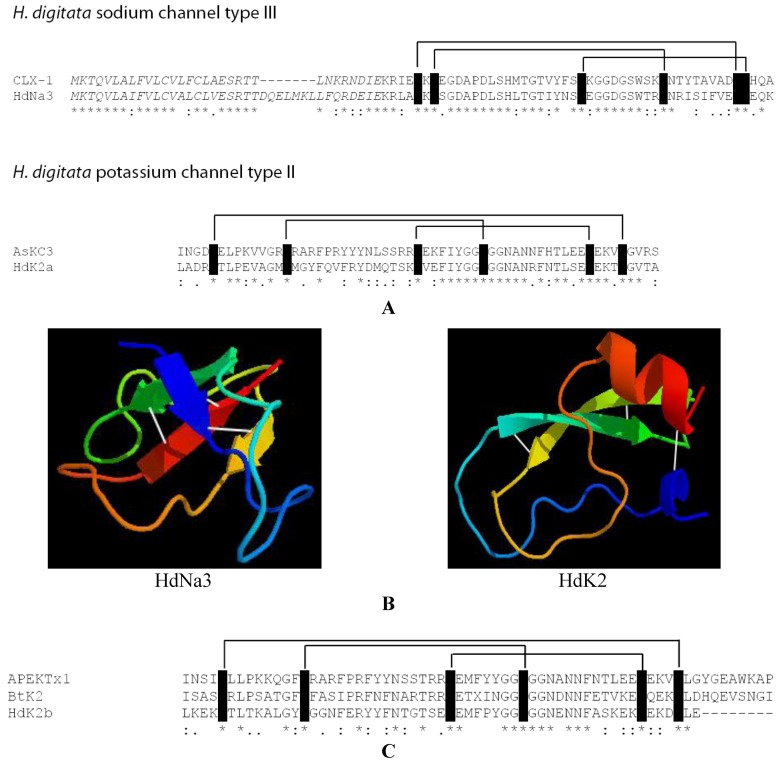
Representative examples of predicted neurotoxin candidates in *H. digitata* transcriptome libraries. (**A**) Recognition of one sodium channel (HdNa3) and one potassium channel (HdK2a) neurotoxin candidates from *H. digitata* based on amino acid sequence alignments. Observed cysteine residues involved in disulfide bridges are indicated. The *N*-terminal leader peptide sequences (italics) are proposed to be cleaved off at the cleavage tandem sequence (KR). (**B**) Structure predictions of the HdNa3 and HdK2a maturepeptide regions. Predictions were made in SWISS-MODEL. The sodium channel neurotoxin predictions contain only β-sheets and loops, in contrast with the potassium channel neurotoxin that also contains an α-helix motif. Disulfide bridges are indicated by white lines between β-sheet motifs. (**C**) Additional two potassium channel neurotoxin candidates from group II, one predicted for *Bolocera* (BtK2) and one for *Hormathia* (HdK2b). 3D structure predictions of both of these type II potassium channel toxins are similar to HdK2a potassium channel neurotoxin from *H. digitata*. Note that star (*) below alignments in (**A**,**C**) indicates identical amino acids. Conserved amino acid changes are indicated by (: or ·).

All possible hits to the query sequences with e-value lower than 1 × 10^−6^ were evaluated (after reciprocal searches to the reviewed SwissProt/UniProtKB protein database) and resulted in 15 hits, 4 for *Bolocera* and 11 for *Hormathia* (Table S2). Another level of stringency was added by allowing only sequences with e-value lower than 1 × 10^−6^ when blasting against the reviewed SwissProt/UniProtKB database. This search finally resulted in four hits, one for *Bolocera* and three for *Hormathia*, the alignments are shown in Figure 3A. An additional four hits to potential neurotoxin candidates were also included in this study (Table S2).

The second approach identifies potential sequences of interest in the digital library based on recognition of conserved domains. The domain architecture is sometimes the only indication to derived protein function, and domain analysis will thus increase the probability of discovering novel compounds. Recognition of conserved domains is also based on homology searches, but here multiple sequence alignment models based on experimentally verified structures make up the basis of the search tools. Most prediction pipelines are designed for single sequence analyses, and thus not suited for NGS data. Only a few studies have applied motif recognition on large-scale data set. Kozlov and co-workers [31,32] have developed a motif recognition program called Single Residue Distribution Analysis (SRDA) where predicted motifs, based on conserved amino acid sites in a certain group of proteins or peptides, are used in scanning of translated EST databases. This method was successfully applied on spider and sea anemone EST databases in the identification of potentially novel neurotoxins. Here, conserved domains were recognized using the NCBI’s Conserved Domain Database (CDD) with the Batch CD-Search interface, which can process up to 100,000 sequence predictions at one time [33]. The CDD input data are amino acid sequences, and nucleotide data have to be translated into the correct reading frames prior to analysis. The complete search results are then compiled into a temporary database, which is downloaded or viewed graphically. The output domain footprint is either shown as specific hits, as domain super families, or as multi-domain models. 

### 2.4. Identification of New Potential Neurotoxin Drug Candidates from Sea Anemones

As a proof-of-concept we applied the protocol in pursuing neurotoxin transcripts in the 454 pyrosequenced transcriptome data from the two cold-water sea anemone species *B. tuediae* and *H. digitata*. Unlike many tropical species these sea anemones are non-symbiotic, meaning they are not associated with a zooxanthellae. Although we cannot exclude co-extraction of RNA from protozoa or microbial species, we find it highly likely that transcripts investigated here originate from the sea anemone tissue. Sodium and potassium channel toxins blast searches (blastx) were performed on our local *Bolocera *and *Hormathia* transcriptome databases, applying published peptide sequences from other Actiniaria as query sequences (Table S1). Most of these toxins are small peptides, less than a hundred amino acids long. Based on the sequence similarity with published sea anemone neurotoxins, we predicted one sodium channel toxin and three potassium channel toxins, which passed our quite stringent criteria for homology prediction (Figure 3A,C). Reciprocal searches against SwissProt/UniProtKB protein database were performed to assure that the blast hits for neurotoxin genes truly represent the best matches for the sequences. One of the classification parameters of sodium channel toxins is the size of the mature toxins, although the number and positions of cysteine residues seem also to be of greater importance [34,35]. It is well known that the 3D structure of neurotoxins is essential for appropriate binding to the specific ion channel and therefore deletions in loop regions might not be vital. The predicted *Hormathia* type III sodium channel toxin (HdNa3) was aligned to the type III homolog from *Calliactis parasitica* (CLX-1). The type III sodium channel toxins are not well defined. The predicted HdNa3 has, however, sequence similarity to the CLX-1 peptide. Surprisingly, no sodium channel toxins were predicted from the *Bolocera* sequence data. This was unexpected since sodium channel toxins are abundant in other sea anemones, and because a sodium channel toxin has previously been reported from *B. tuediae* [36]. 

Transcripts representing type II class of potassium channel toxins were predicted from both *B. tuediae* and *H. digitata *(Figure 3A,C). Type II toxins appear to be well conserved both regarding sequence and structure prediction. HdK2a aligns well to the type II toxin from *Anemonia sulcata* (AsKC3) (Figure 3A), two additional toxins BtK2 and HdK2b align both well to the type II toxin from *Anthopleura elegantissima* (APEKTx1). 

Some toxins are represented as precursors that include an *N*-terminal signal peptide (Figure 3A). Peptide cleavage is usually initiated at a cleavage tandem site (Lys-Arg) leaving a mature peptide at the *C*-terminal part [37]. With the exception of a few conserved domains, neurotoxins generally have limited sequence conservation. The predicted neurotoxin sequences were therefore structure determined by SWISS-MODEL in order to establish conserved structural motifs, and thereby support the sequence predictions. SWISS-MODEL 3D predictions resulted in four β-strands configurations of HdNa3 (Figure 3B). This is in agreement with the information stating that most sodium channel toxins seem not to possess α-helix motifs [22]. The potassium channel toxin, HdK2 (Figure 3B), was predicted by SWISS-MODEL to possess a *C*-terminal, and also small *N*-terminal α-helix, presence of helices is comparable to other type II potassium channel toxins [27].

An additional 15 toxin candidates were predicted using lower stringency settings, 4 for *B. tuediae* and 11 for *H. digitata* (Table S2). These would be also included in possible experimental follow-up studies. However the digital marine bioprospecting approach introduced here serves only for bioinformatic purposes to identify possible homology variants of known proteins by mining next generation sequencing data. 

Four of these additional toxin candidates showed high similarity to proteins with Kunitz-type domain (AXPI and Kunitz/BPTI-like toxin). AXPI protein was already previously shown to have sequence similarity to type II potassium channel toxins (AsKC1-3) and was proven to belong to the Kunitz-type family [38]. Proteins from this family have high sequence similarity, concerning especially the position of Cysteine residues, and they share both protease, as well as ion channel inhibitory activity. AXPI protein is also predicted to be structurally similar to these proteins, a sign also very important for neurotoxins, although its possible ion channel inhibitory activity has not been proven yet. 

Potential neurotoxin sequences were also predicted by recognizing conserved domains (Table 2). We obtained 144 and 206 neurotoxin candidate transcripts from *B. tuediae* and *H. digitata*, respectively. The output result from the Batch CD-Search tool assigned 131 (*B. tuediae*) and 229 (*H. digitata*) superfamily queries with 151 and 267 positive domain hits, respectively, and a total of nine unique domains (Table 2, S3 and S4). The most widespread conserved domain was the BPT1/Kunitz superfamily, originally a serine protease inhibitor that has gained a new function as an ion channel blocking toxin [39]. Other serine protease inhibitor domains were also recognized, together with domains characterized by disulfide bridges. A significant fraction of the transcripts was found to contain more than one conserved recognized domain.

**Table 2:** Conserved domain recognition.

CDD, input and output ^a^	*B. tuediae*	*H. digitata*
Query amino acid sequences	864	1236
Queries with domain hits	131	229
Total number of domain hits	151	267
**Superfamilies**		
KU (Kunitz-type)	135	211
Toxin4	-	23
KAZAL_FS	6	23
Antistatin	6	-
WAP	1	3
TY	2	1
ShK	-	1
VMA21-like	-	1
NTR	-	1

^a^ Conserved domain recognition in transcriptome data from *B. tuediae* and *H. digitata*. A neurotoxin-enriched portion of the 454 transcriptome raw reads was translated into six reading frames and ran through the NCBI’s Conserved Domain Databases (CDD). Recognized superfamily domains included: KU—Kunitz type toxins (serine proteinase inhibitor); Toxin4—sea anemone neurotoxin; KAZAL_FS—serine protease inhibitor, Antistatin—serine protease inhibitor; WAP—whey acidic protein-type four-sulfide core domains; TY—thyroglobin type I; ShK—three disulfide bridges, potassium channel inhibitor; VMA21-like—two potential transmembrane helicos; NTR-like—beta barrel.

## 3. Experimental Section

### 3.1. Sampling and RNA Extraction

The cold-water sea anemones *B. tuediae* (Order Actiniaria; Family Actiniidae) and *H. digitata* (Order Actiniaria; Family Hormathiidae) were collected 2009-10-01 in Tromsø, Norway (69°41′ N; 18°56′ E) at 25 m depth using scuba diving (Figure 1A). RNA was extracted by crushing fresh tissue from body wall and tentacles in TRIzol using a Precellys lysis homogenizer (Stretton Scientific, Stretton, UK) to ensure identical sample handling before extraction [40]. 0.2× volume of chloroform was added, incubated on ice for 20 min, centrifuged and the water phase was transferred to a new tube. The RNA was precipitated in isopropanol at 4 °C and the pellet washed with 75% ethanol before the RNA was rehydrated in water. For some of the samples an additional phenol/chloroform extraction was performed, and subsequently RNA was precipitated in ethanol. 

### 3.2. Large Scale Sequencing

Transcriptome sequencing of fragment libraries was performed by the 454 pyrosequencing platform at Eurofins MWG Operon (Germany). Approximately 10 µg total RNA from each species was shipped to Germany. Poly(A)^+^ RNA was prepared by Eurofins MWG Operon, first strand cDNA was synthesized applying random hexamers, with successive ligation of 5′ and 3′ adaptors. PCR amplification was performed with a proof-reading enzyme. Normalization was carried out by denaturation and renaturation of the cDNA, with subsequent removal of ds-cDNA before ss-cDNA PCR amplification. The cDNA was size fractionated (500–700 bp) by elution of preparative agarose gels, subjected to shotgun library preparation and subsequent GS FLX Titanium sequencing. All the handling of the samples after RNA isolation, including cDNA library preparation, was done by Eurofins MWG Operon.

### 3.3. Assembly, Mapping and Annotation

The contig assemblies were performed as a service by the Eurofins MWG Operon. Quality-filtering of the reads was done by Roche/454 sequencer software when performing the base calling. The sequences were additionally trimmed, the key tag (TCAG) at the 5′ end and low quality and adapter sequences were removed from the sequences before assembly by MIRA Assembler software. Only reads ≥40 bp were considered for the assembly by MIRA Assembler. Assembled contigs and single reads were run through Blast2GO [41], they were BLASTed, mapped and annotated. The transcripts were grouped, based on their potential function and visualized in bar charts applying CateGOrizer [42] and Microsoft Excel. Additional statistical data were extracted from Blast2GO. The transcriptome data were collected in two local databases and blastx searches were performed on the contigs and single reads using relevant published anthozoan sequences as queries with a threshold value of e = 1 × 10^−6^. Furthermore, CLC Genomic Workbench [43] was applied in the mapping of toxins. Different stringencies were used during the neurotoxin searches and also additional reciprocal searches against second, more comprehensive SwissProt/UniProtKB protein database were performed to verify the candidate toxin hits.

### 3.4. Structure and Domain Predictions

Multiple alignments were made in ClustalW2 [44], and the mature protein length for the aligned known toxins was determined using Protein Knowledgebase (SwissProt/UniProtKB). Cysteine residues in the alignments were highlighted by black colour and the disulphide bridges were marked by lines. 3D structure predictions were performed by SWISS-MODEL [45,46] using translated amino acid sequences. The 3D structures were exported in .pdb format and visualized in PyMol Viewer [47] as a cartoon with α-helices and β-sheets with a colour transition from red to blue, *C*- to *N*-terminal. Additionally, disulphide bridges were marked according to the sequence alignments. For better resolution, figures were exported and run in POV-Ray [48].

A collection of sequences were also translated in all six reading frames applying the Six Frame Translation tool from Max-Planck Institute for Developmental Biology [49], and run through the Batch Conserved Domain Database [33] with default settings. For the purpose of this analysis, the transcriptome sequences were first enriched with potential neurotoxin transcripts by performing a low stringency (e = 1 × 10^−2^) blastx homology search applying 78 annotated neurotoxins (Table S1) as queries and these candidate sequences were translated into amino acid sequences in all six reading frames before being introduced to the Batch CD-Search tool.

## 4. Conclusion

Whole transcriptome profiling based on deep sequencing technologies has revolutionized the field of gene expression. In this study we report high-throughput 454 pyrosequencing to generate draft assemblies of adult polyp transcriptomes in two distantly related cold-water sea anemone species. Interestingly, the transcriptome profiles were highly similar between species. The datasets were stored as digital libraries from which desired genes and gene motifs were recognized. As a proof-of-concept we performed searches for neurotoxins by following two different approaches; homology searches and conserved domain recognition. Homology searches obtained precise hits determined by the stringency of the search, while functional domain annotation increased the chances of finding novel molecules with a certain function despite limited recognition at the nucleotide level. The fact that we identified four highly similar and 15 additional new neurotoxin peptide candidates from the *Bolocera* and *Hormathia* transcriptomes confirms the potential of digital bioprospecting. The next step in fulfilling the protocol is to include high-throughput functional analysis of candidate peptide/proteins in an appropriate experimental setting. The recent developments in array-based protein function analyses are very promising and have resulted in cell-free protein synthesis and high-density protein array platforms [50,51]. Combining these fields of biological science (bioinformatics, transcriptomics and proteomics) will create a powerful complementary approach to marine bioprospecting, which require only minute amounts of sample materials in the discovery and investigation of new protein-based drug candidates.

## Supplementary Files

Supplementary File 1: PDF-Document (PDF, 75 KB)
