# Supplementary material for: Digital Marine Bioprospecting: Mining New Neurotoxin Drug Candidates from the Transcriptomes of Cold-Water Sea Anemones
Source: Mar Drugs. 2012 Oct 18;10(10):2265–79. doi: 10.3390/md10102265 (PMC3497022; doi:10.3390/md10102265)
Supplement: Supplementary File 1: — PDF-Document (PDF, 75 KB) [file marinedrugs-10-02265-s001.pdf]

Supplementary Materials

Table S1. 78 neurotoxins from 26 sea anemone species were downloaded from the NCBI database. 54 sodium channel toxins, 16 potassium channel toxins and 7 additional peptide toxins were applied as query sequences in tBlastn homology searches against two local transcriptome databases from the sea anemone species *Bolocera tuediae* and *Hormathia digitata*.

Toxin type	Name	Size	GI number	Species
Na+ channel neurotoxins Type I	Ae I	54aa	22002011	*Actinia equina*
Na+ channel neurotoxins Type I	AETX-1	47aa	57012555	*Anemonia erythraea*
Na+ channel neurotoxins Type I	AFT-I	47aa	136522	*Anthopleura fuscoviridis*
Na+ channel neurotoxins Type I	AFT-II	48aa	136524	*Anthopleura fuscoviridis*
Na+ channel neurotoxins Type I	Am-III	47aa	57013132	*Antheopsis maculata*
Na+ channel neurotoxins Type I	Anthopleurin-A (ApA)	49aa	136530	*Anthopleura xanthogrammica*
Na+ channel neurotoxins Type I	Anthopleurin-B (ApB)	49aa	136531	*Anthopleura xanthogrammica*
Na+ channel neurotoxins Type I	Anthopleurin-C (ApC)	47aa	136532	*Anthopleura elegantissima*
Na+ channel neurotoxins Type I	APE 1-1	47aa	97217055	*Anthopleura elegantissima*
Na+ channel neurotoxins Type I	APE 1-2	47aa	97217059	*Anthopleura elegantissima*
Na+ channel neurotoxins Type I	APE 2-1	47aa	97217062	*Anthopleura elegantissima*
Na+ channel neurotoxins Type I	APE 2-2	47aa	97217066	*Anthopleura elegantissima*
Na+ channel neurotoxins Type I	ATX-1	46aa	136521	*Anemonia sulcata*
Na+ channel neurotoxins Type I	ATX-2	47aa	136523	*Anemonia sulcata*
Na+ channel neurotoxins Type I	ATX-5	46aa	136529	*Anemonia sulcata*
Na+ channel neurotoxins Type I	BcIII	48aa	47117401	*Bunodosoma caissarum*
Na+ channel neurotoxins Type I	Bcg	47aa	292630904	*Bunodosoma cangicum*
Na+ channel neurotoxins Type I	Bg-II	48aa	97217045	*Bunodosoma granuliferum*
Na+ channel neurotoxins Type I	Bg-III	48aa	97217050	*Bunodosoma granuliferum*
Na+ channel neurotoxins Type I	Cangitoxin (CGTX)	48aa	18202429	*Bunodosoma cangicum*
Na+ channel neurotoxins Type I	Cangitoxin II	48aa	193806548	*Bunodosoma cangicum*
Na+ channel neurotoxins Type I	Cangitoxin III	48aa	193806549	*Bunodosoma cangicum*
Na+ channel neurotoxins Type I	CgNa	47aa	119367403	*Condylactis gigantea*
Na+ channel neurotoxins Type I	Cp-1	47aa	306756325	*Condylactis passiflora*
Na+ channel neurotoxins Type I	Gigantoxin II (GigtII)	44aa	57012800	*Stichodactyla gigantea*
Na+ channel neurotoxins Type I	Hk2	47aa	158706398	*Anthopleura* sp. 'Zhanjiang'
Na+ channel neurotoxins Type I	Hk7	47aa	158706399	*Anthopleura* sp. 'Zhanjiang'
Na+ channel neurotoxins Type I	Hk8	47aa	158706400	*Anthopleura* sp. 'Zhanjiang'
Na+ channel neurotoxins Type I	Hk16	47aa	158706397	*Anthopleura* sp. 'Zhanjiang'
Na+ channel neurotoxins Type I	PCR1	47aa	158706390	*Anthopleura xanthogrammica*
Na+ channel neurotoxins Type I	PCR2	47aa	158706391	*Anthopleura xanthogrammica*
Na+ channel neurotoxins Type I	PCR3	47aa	158706392	*Anthopleura xanthogrammica*
Na+ channel neurotoxins Type I	PCR4	47aa	158706393	*Anthopleura xanthogrammica*
Na+ channel neurotoxins Type I	PCR5	47aa	158706394	*Anthopleura xanthogrammica*
Na+ channel neurotoxins Type I	PCR6	49aa	158706395	*Anthopleura xanthogrammica*
Na+ channel neurotoxins Type I	PCR7	49aa	158706396	*Anthopleura xanthogrammica*
Na+ channel neurotoxins Type I	Rc-1	47aa	158706401	*Heteractis crispa*
Na+ channel neurotoxins Type II	Delta-thalatoxin-Ca1a	49aa	322518481	*Cryptodendrum adhaesivum*
Na+ channel neurotoxins Type II	Delta-thalatoxin-Ta1a	49aa	322518534	*Thalassianthus aster*
Na+ channel neurotoxins Type II	Delta-thalatoxin-Hh1x	49aa	322518561	*Heterodactyla hemprichi*

Table S1. *Cont.*

Na+ channel neurotoxins Type II	Halcurin	47aa	158705827	*Halcurias carlgreni*
Na+ channel neurotoxins Type II	Gigantoxin III (GigtIII)	48aa	57012798	*Stichodactyla gigantea*
Na+ channel neurotoxins Type II	Nv3-3	47aa	306756340	*Nematostella vectensis*
Na+ channel neurotoxins Type II	RpII	48aa	136525	*Radianthus paumotensis*
Na+ channel neurotoxins Type II	RpIII	48aa	136528	*Radianthus paumotensis*
Na+ channel neurotoxins Type II	RTX-I (RmI)	48aa	401226	*Heteractis crispa*
Na+ channel neurotoxins Type II	RTX-II (Rm2)	48aa	401227	*Heteractis crispa*
Na+ channel neurotoxins Type II	RTX-III (Rm3)	48aa	401228	*Heteractis crispa*
Na+ channel neurotoxins Type II	RTX-V (Rm5)	47aa	401230	*Heteractis crispa*
Na+ channel neurotoxins Type II	SHP-I (ShI)	48aa	136512	*Stichodactyla helianthus*
Na+ channel neurotoxins Type III	Calitoxin-1 (CLX-1)	46aa	1352093	*Calliactis parasitica*
Na+ channel neurotoxins Type III	Calitoxin-2 (CLX-2)	46aa	1352094	*Calliactis parasitica*
Na+ channel neurotoxins No assigned type	ATX-III	27aa	136526	*Anemonia sulcata*
Na+ channel neurotoxins No assigned type	PA-TX	31aa	136527	*Entacmaea quadricolor*
K+ channel neurotoxins Type I	Aek	36aa	6226209	*Actinia equina*
K+ channel neurotoxins Type I	AETX-K	34aa	121953299	*Anemonia erythraea*
K+ channel neurotoxins Type I	Bgk	37aa	2506190	*Bunodosoma granuliferum*
K+ channel neurotoxins Type I	HmK	35aa	97217083	*Heteractis magnifica*
K+ channel neurotoxins Type I	Kaliseptin (AsKS)	36aa	55976776	*Anemonia sulcata*
K+ channel neurotoxins Type I	ShK	35aa	136540	*Stichodactyla helianthus*
K+ channel neurotoxins Type II	Kalicludine-1 (AsKC1)	58aa	1181912	*Anemonia sulcata*
K+ channel neurotoxins Type II	Kalicludine-2 (AsKC2)	58aa	1181913	*Anemonia sulcata*
K+ channel neurotoxins Type II	Kalicludine-3 (AsKC3)	59aa	1181914	*Anemonia sulcata*
K+ channel neurotoxins Type III	Am-2	46aa	57013134	*Antheopsis maculata*
K+ channel neurotoxins Type III	APETx1	42aa	47605396	*Anthopleura elegantissima*
K+ channel neurotoxins Type III	APETx2	42aa	47605397	*Anthopleura elegantissima*
K+ channel neurotoxins Type III	BDS-1	43aa	114907	*Anemonia sulcata*
K+ channel neurotoxins Type III	BDS-2	43aa	25008168	*Anemonia sulcata*
K+ channel neurotoxins No assigned type	APEKTx1	65aa	338817561	*Anthopleura elegantissima*
K+ channel neurotoxins No assigned type	SHTX-3	62aa	206557800	*Stichodactyla haddoni*
Other peptide toxins	Am-I	27aa	57013133	*Antheopsis maculata*
Other peptide toxins	Acrorhagin I	50aa	90111958	*Actinia equina*
Other peptide toxins	Acrorhagin Ia	52aa	90111957	*Actinia equina*
Other peptide toxins	Acrorhagin II	44aa	90111960	*Actinia equina*
Other peptide toxins	Acrorhagin IIa	44aa	90111959	*Actinia equina*
Other peptide toxins	AETX-II	59aa	57012556	*Anemonia erythraea*
Other peptide toxins	AETX-III	59aa	57012557	*Anemonia erythraea*
Other peptide toxins	Gigantoxin I (GigtI)	48aa	57012799	*Stichodactyla gigantea*

Table S2. Presented here are additional candidate neurotoxins, which have not passed our stringent criteria to be submitted as the best hits for potential new sea anemone neurotoxins. In the table are visualized complete sequences together with appropriate species and the best BLAST neurotoxin hits. E-values for the searches against SwissProt/UniProtKB database are also shown. All sequences with e > 1 × 10^{-6} were not considered in our further analyses; alignments to AXPI and Kunitz/BPTI-like toxins are exceptions, since their ion channel inhibitory activity was not yet confirmed, despite their sequence and structural homology to the known neurotoxins.

Sequence	Specie	Neurotoxin similarity	e-value
IVYSVESQNTYCLDDCFGDLSSCQLSDRCKSPATCAVCTSDYQNCTPKCS	*B. tuediae*	Acrorhagin I	6 × 10^{-5}
ATSCRCVINHYFRHGTFWFGRRSCPSGHGYNKSCFRWFGRCCVKF	*B. tuediae*	APETx2	1.5 × 10^{-2}
TSCRCVINHYFRHGTFWFGRRSCPSGHGYNKSCFRWFGRCC	*B. tuediae*	APETx2	1 × 10^{-2}
NCLLPKVVGPCRAAMPRFYYNSGNCEQFTYGGCDGNANNFNTEEECKKAC	*B. tuediae*	AXPI	2 × 10^{-27}
MSRLLIIFIVVTVVGCVMGKPNEGVVRDVEKRDGLRCLCDDQRTEGIKWLFNCRSGWTQCGSGIMKCCHQ	*H. digitata*	Gigt II	2 × 10^{-3}
MNRLLIVLIGVTLVALATGVRELKDESVLQKRAGTPCDCDTGRYGGEDGIHWAFGCPSDEWKLCGTSSHYLIQIPCC	*H. digitata*	Gigt II	2 × 10^{-4}
DCKDKAYNCDRLQSACRTSWNIKTYWCKKTCGTC	*H. digitata*	AETX K	4.8
MKRLYFLILAALVIVSHVEGQCSKDCYGTFQTCYFGCTAITGPKCFNDCQSVYAVCLNTGC	*H. digitata*	Acrorhagin I	2 × 10^{-3}
LVHTARAADAGFCREECFSRQQLCMFSKNNCYNKKSCMSCVKSFSPCLMGC	*H. digitata*	Acrorhagin I	6 × 10^{-5}
ICKQPDYPGPCNSMRKRWFFDSHTGKCNSFNYGGCYGNENNFATQDLCHQRC	*H. digitata*	Kunitz/BPTI-like toxin	5 × 10^{-5}
CFLPRTIGPCREYIPRFYYNRETRRCEAFGYGGCEGNANNYNTKEECEQKC	*H. digitata*	AXPI	2 × 10^{-22}
ATSCRCVINHYFRHGTFWFGRRSCPSGHGYNKSCFRWFGRCC	*H. digitata*	APETx2	1.2 × 10^{-2}
GLRCLCDDQRTEGIKWLFNCRSGWTQCGSGIMKCCHQ	*H. digitata*	PCR4	3 × 10^{-2}
CLLPKVTGPCRAAMPRYYYNSKSGNCEQFTYGGCDGNANNFATPEKCKAAC	*H. digitata*	AXPI	5 × 10^{-27}
CKQPDYPGPCNSMRKRWFFDSHTGKCNSFNYGGCYGNENNFATQDLCHQRC	*H. digitata*	Kunitz/BPTI-like toxin	5 × 10^{-5}

Table S3. Conserved domain hits for transcripts from *B. tuediae*.

Query	PSSM-ID	From	To	E-Value	Bitscore	Accession	Short name
Q#7 - >2464__F6AHOR30	206837	80	130	1.15669e-11	56.4395	cl00101	KU superfamily
Q#7 - >2464__F6AHOR30	206837	35	78	4.47599e-07	43.3427	cl00101	KU superfamily
Q#15 - >4998__F6AHOR30	206837	9	62	1.77243e-23	88.0258	cl00101	KU superfamily
Q#19 - >8165__F6AHOR30	145792	3	28	5.22364e-05	37.6255	cl10597	Antistasin superfamily
Q#20 - >8165__F6AHOR30	206837	53	86	4.46384e-12	57.5951	cl00101	KU superfamily
Q#26 - >8650__F6AHOR30	206837	36	89	5.51717e-17	70.6919	cl00101	KU superfamily
Q#26 - >8650__F6AHOR30	145792	6	31	0.000612912	34.5439	cl10597	Antistasin superfamily
Q#31 - >13666__F6AHOR3	206837	19	72	1.66883e-15	66.8399	cl00101	KU superfamily
Q#38 - >23218__F6AHOR3	206837	74	117	1.00642e-06	42.1871	cl00101	KU superfamily
Q#38 - >23218__F6AHOR3	206834	20	60	1.11354e-06	41.8163	cl00097	KAZAL_FS superfamily
Q#44 - >23234__F6AHOR3	206837	99	142	1.97856e-06	41.8019	cl00101	KU superfamily
Q#44 - >23234__F6AHOR3	206834	45	85	2.38826e-06	41.4311	cl00097	KAZAL_FS superfamily
Q#49 - >23491__F6AHOR3	206837	41	91	4.5733e-17	71.4623	cl00101	KU superfamily
Q#62 - >29752__F6AHOR3	206837	49	99	2.94184e-16	68.7659	cl00101	KU superfamily
Q#75 - >31486__F6AHOR3	206837	76	121	3.92096e-17	71.0771	cl00101	KU superfamily
Q#75 - >31486__F6AHOR3	206837	1	33	4.84822e-07	42.9575	cl00101	KU superfamily
Q#89 - >45378__F6AHOR3	206837	55	105	7.09721e-16	67.9955	cl00101	KU superfamily
Q#92 - >45938__F6AHOR3	206837	26	76	7.06284e-18	73.3883	cl00101	KU superfamily
Q#110 - >51059__F6AHOR3	206837	22	74	1.54493e-21	83.0183	cl00101	KU superfamily
Q#115 - >56458__F6AHOR3	206837	92	111	0.000934937	34.967	cl00101	KU superfamily
Q#116 - >56458__F6AHOR3	206837	111	137	5.6343e-07	43.7279	cl00101	KU superfamily
Q#121 - >57768__F6AHOR3	206837	25	75	5.68647e-19	76.4699	cl00101	KU superfamily
Q#129 - >59553__F6AHOR3	206837	55	107	4.99077e-19	76.4699	cl00101	KU superfamily
Q#133 - >66372__F6AHOR3	206837	1	37	1.22138e-12	59.1359	cl00101	KU superfamily
Q#141 - >66552__F6AHOR3	206837	9	62	4.47452e-15	66.0695	cl00101	KU superfamily
Q#146 - >70813__F6AHOR3	145792	51	76	0.000161696	36.8551	cl10597	Antistasin superfamily
Q#147 - >70813__F6AHOR3	206837	81	134	2.01819e-20	80.7071	cl00101	KU superfamily
Q#151 - >71226__F6AHOR3	206837	1	51	2.32753e-18	74.5439	cl00101	KU superfamily
Q#159 - >74976__F6AHOR3	206837	48	98	3.72983e-17	71.4623	cl00101	KU superfamily
Q#165 - >77553__F6AHOR3	206837	76	121	3.92096e-17	71.0771	cl00101	KU superfamily
Q#165 - >77553__F6AHOR3	206837	1	33	4.84822e-07	42.9575	cl00101	KU superfamily
Q#171 - >81167__F6AHOR3	206837	1	44	5.64265e-14	62.9879	cl00101	KU superfamily
Q#176 - >85155__F6AHOR3	206837	22	74	6.11999e-23	87.2554	cl00101	KU superfamily
Q#182 - >95260__F6AHOR3	206837	33	85	2.54859e-18	74.9291	cl00101	KU superfamily
Q#188 - >95838__F6AHOR3	206837	82	134	2.88159e-11	55.2839	cl00101	KU superfamily
Q#194 - >97653__F6AHOR3	206837	26	76	1.28095e-12	59.1359	cl00101	KU superfamily
Q#200 - >104626__F6AHOR	206837	79	115	4.51544e-13	60.6767	cl00101	KU superfamily
Q#207 - >106141__F6AHOR	206837	45	98	3.07408e-21	82.6331	cl00101	KU superfamily
Q#211 - >106345__F6AHOR	206837	42	95	1.94946e-14	64.1435	cl00101	KU superfamily
Q#211 - >106345__F6AHOR	206837	1	40	4.39688e-06	40.7449	cl00101	KU superfamily
Q#218 - >113893__F6AHOR	206837	40	88	1.27895e-19	77.6255	cl00101	KU superfamily
Q#224 - >126829__F6AHOR	206837	49	99	1.11768e-17	73.3883	cl00101	KU superfamily
Q#231 - >132715__F6AHOR	206837	58	107	4.57007e-18	73.3883	cl00101	KU superfamily
Q#242 - >157576__F6AHOR	206837	22	66	2.55684e-16	69.9215	cl00101	KU superfamily
Q#249 - >160540__F6AHOR	206837	22	74	5.37958e-24	89.9518	cl00101	KU superfamily
Q#253 - >161209__F6AHOR	206837	50	100	4.26916e-18	74.5439	cl00101	KU superfamily
Q#261 - >161620__F6AHOR	206837	46	99	4.10535e-14	63.3731	cl00101	KU superfamily
Q#261 - >161620__F6AHOR	206837	1	44	4.78347e-07	43.3427	cl00101	KU superfamily
Q#267 - >161886__F6AHOR	206837	36	86	9.76864e-18	73.3883	cl00101	KU superfamily

Table S3. *Cont.*

Q#278 - >175478__F6AHOR	206837	57	105	1.75054e-20	79.9367	cl00101	KU superfamily
Q#284 - >178435__F6AHOR	206837	1	45	3.39143e-16	69.1511	cl00101	KU superfamily
Q#289 - >178675__F6AHOR	206837	38	78	2.54386e-18	74.5439	cl00101	KU superfamily
Q#291 - >178675__F6AHOR	206837	1	23	3.76934e-08	46.4243	cl00101	KU superfamily
Q#301 - >182000__F6AHOR	206837	1	27	4.20534e-08	46.4243	cl00101	KU superfamily
Q#308 - >183748__F6AHOR	206837	61	114	4.77419e-14	62.9879	cl00101	KU superfamily
Q#308 - >183748__F6AHOR	206837	16	59	5.84551e-07	43.3427	cl00101	KU superfamily
Q#315 - >190363__F6AHOR	206837	56	109	1.21028e-17	73.0031	cl00101	KU superfamily
Q#325 - >193381__F6AHOR	206837	118	156	9.26395e-08	45.2687	cl00101	KU superfamily
Q#338 - >200100__F6AHOR	145792	5	30	1.36368e-05	39.1663	cl10597	Antistasin superfamily
Q#339 - >200100__F6AHOR	206837	35	85	1.14613e-19	78.0107	cl00101	KU superfamily
Q#345 - >201434__F6AHOR	206837	48	98	6.47289e-17	71.4623	cl00101	KU superfamily
Q#349 - >204196__F6AHOR	206837	25	75	4.55386e-20	79.5515	cl00101	KU superfamily
Q#356 - >213209__F6AHOR	206837	2	44	2.62284e-16	69.5363	cl00101	KU superfamily
Q#362 - >220408__F6AHOR	206837	44	92	8.48127e-20	78.7811	cl00101	KU superfamily
Q#367 - >223700__F6AHOR	206837	19	72	2.51976e-15	66.4547	cl00101	KU superfamily
Q#374 - >224991__F6AHOR	206837	49	99	3.25071e-17	71.8475	cl00101	KU superfamily
Q#379 - >225260__F6AHOR	206837	118	156	9.26395e-08	45.2687	cl00101	KU superfamily
Q#385 - >225321__F6AHOR	206837	83	136	1.50182e-11	56.0543	cl00101	KU superfamily
Q#399 - >231964__F6AHOR	206837	18	62	1.22149e-15	67.9955	cl00101	KU superfamily
Q#404 - >240118__F6AHOR	206837	104	143	2.44712e-08	47.1947	cl00101	KU superfamily
Q#427 - >246912__F6AHOR	206834	57	97	8.92126e-07	42.5867	cl00097	KAZAL_FS superfamily
Q#427 - >246912__F6AHOR	206837	111	147	8.12925e-05	37.6634	cl00101	KU superfamily
Q#427 - >246912__F6AHOR	206798	1	24	0.00612266	32.4348	cl00043	CCP superfamily
Q#435 - >249696__F6AHOR	206837	22	75	6.74069e-21	81.4775	cl00101	KU superfamily
Q#440 - >253934__F6AHOR	206837	130	171	8.11216e-14	62.6027	cl00101	KU superfamily
Q#445 - >260505__F6AHOR	206837	23	76	1.3827e-21	83.4035	cl00101	KU superfamily
Q#452 - >268222__F6AHOR	206837	38	88	6.18423e-18	74.1587	cl00101	KU superfamily
Q#458 - >269325__F6AHOR	206837	97	146	5.53361e-18	73.7735	cl00101	KU superfamily
Q#458 - >269325__F6AHOR	206837	1	54	9.51654e-17	70.6919	cl00101	KU superfamily
Q#464 - >271769__F6AHOR	206837	58	104	2.81782e-13	61.0619	cl00101	KU superfamily
Q#464 - >271769__F6AHOR	145792	28	53	0.0011122	34.5439	cl10597	Antistasin superfamily
Q#469 - >274477__F6AHOR	206837	35	85	8.05198e-16	68.3807	cl00101	KU superfamily
Q#476 - >275702__F6AHOR	206837	126	158	7.91546e-08	45.6539	cl00101	KU superfamily
Q#477 - >275702__F6AHOR	206837	61	104	7.34039e-07	43.3427	cl00101	KU superfamily
Q#477 - >275702__F6AHOR	206834	7	47	7.38092e-07	42.9719	cl00097	KAZAL_FS superfamily
Q#482 - >278612__F6AHOR	206837	86	128	1.46195e-12	59.1359	cl00101	KU superfamily
Q#487 - >281690__F6AHOR	206837	82	100	0.00233342	33.7767	cl00101	KU superfamily
Q#488 - >281690__F6AHOR	206837	56	81	5.32912e-06	41.0315	cl00101	KU superfamily
Q#494 - >284423__F6AHOR	206837	7	60	8.85584e-20	79.1663	cl00101	KU superfamily
Q#494 - >284423__F6AHOR	206863	84	133	3.87357e-13	61.2092	cl00150	TY superfamily
Q#501 - >300003__F6AHOR	206837	19	72	4.91268e-19	76.8551	cl00101	KU superfamily
Q#506 - >304258__F6AHOR	206834	7	47	4.64229e-07	43.3571	cl00097	KAZAL_FS superfamily
Q#506 - >304258__F6AHOR	206837	61	104	7.26437e-07	42.9575	cl00101	KU superfamily
Q#506 - >304258__F6AHOR	206837	106	136	2.2076e-05	39.1695	cl00101	KU superfamily
Q#518 - >311446__F6AHOR	206868	68	90	0.00687527	32.3411	cl00156	WAP superfamily
Q#525 - >311642__F6AHOR	206837	1	36	1.76392e-10	52.9727	cl00101	KU superfamily
Q#525 - >311642__F6AHOR	206863	51	98	2.92587e-09	50.0384	cl00150	TY superfamily
Q#542 - >328359__F6AHOR	206837	36	89	4.32946e-17	71.0771	cl00101	KU superfamily
Q#542 - >328359__F6AHOR	145792	6	31	0.000758282	34.5439	cl10597	Antistasin superfamily

Table S3. *Cont.*

Q#548 - >330186__F6AHOR	206837	73	126	3.03194e-17	71.8475	cl00101	KU superfamily
Q#559 - >337501__F6AHOR	206837	31	83	2.44778e-19	78.0107	cl00101	KU superfamily
Q#566 - >340377__F6AHOR	206837	40	90	9.1323e-17	70.3067	cl00101	KU superfamily
Q#571 - >349362__F6AHOR	206837	61	107	8.88196e-18	73.3883	cl00101	KU superfamily
Q#577 - >351014__F6AHOR	206837	2	33	2.72017e-11	56.8247	cl00101	KU superfamily
Q#591 - >365577__F6AHOR	206837	17	61	2.06306e-14	64.5287	cl00101	KU superfamily
Q#607 - >376827__F6AHOR	206837	49	84	4.13996e-10	52.2023	cl00101	KU superfamily
Q#615 - >382259__F6AHOR	206837	58	106	1.37088e-20	80.3219	cl00101	KU superfamily
Q#619 - >389206__F6AHOR	206837	19	63	2.49605e-16	69.9215	cl00101	KU superfamily
Q#625 - >394231__F6AHOR	206837	81	99	0.000588834	35.7027	cl00101	KU superfamily
Q#626 - >394231__F6AHOR	206837	49	80	1.28308e-08	47.9651	cl00101	KU superfamily
Q#632 - >401702__F6AHOR	206837	87	130	1.76416e-06	41.8019	cl00101	KU superfamily
Q#632 - >401702__F6AHOR	206834	33	73	1.90485e-06	41.8163	cl00097	KAZAL_FS superfamily
Q#637 - >417378__F6AHOR	206837	1	42	4.28802e-16	68.7659	cl00101	KU superfamily
Q#644 - >418224__F6AHOR	206837	32	82	8.30364e-22	85.3294	cl00101	KU superfamily
Q#645 - >418224__F6AHOR	206837	1	21	8.35841e-07	44.1131	cl00101	KU superfamily
Q#650 - >423007__F6AHOR	206837	33	83	2.27701e-17	72.6179	cl00101	KU superfamily
Q#655 - >429340__F6AHOR	206837	69	120	6.15555e-13	60.2915	cl00101	KU superfamily
Q#663 - >434736__F6AHOR	206837	23	74	1.31599e-19	78.7811	cl00101	KU superfamily
Q#668 - >437132__F6AHOR	206837	24	77	6.80049e-17	69.9215	cl00101	KU superfamily
Q#675 - >441767__F6AHOR	206837	55	99	6.00893e-14	62.9879	cl00101	KU superfamily
Q#679 - >454132__F6AHOR	206837	45	85	9.20785e-15	65.2991	cl00101	KU superfamily
Q#687 - >455427__F6AHOR	206837	36	86	2.13653e-17	73.0031	cl00101	KU superfamily
Q#691 - >463355__F6AHOR	206837	69	93	7.41117e-08	45.6539	cl00101	KU superfamily
Q#699 - >464645__F6AHOR	206837	66	119	4.63753e-17	71.0771	cl00101	KU superfamily
Q#711 - >470526__F6AHOR	206837	1	24	3.14796e-09	51.4319	cl00101	KU superfamily
Q#723 - >477373__F6AHOR	206837	27	80	2.80264e-13	62.6027	cl00101	KU superfamily
Q#728 - >479079__F6AHOR	206837	41	75	1.88113e-09	50.6615	cl00101	KU superfamily
Q#728 - >479079__F6AHOR	206837	108	134	3.92967e-06	41.5153	cl00101	KU superfamily
Q#729 - >479079__F6AHOR	206837	75	93	6.68073e-05	38.3991	cl00101	KU superfamily
Q#734 - >481987__F6AHOR	206837	94	134	7.37123e-13	59.9063	cl00101	KU superfamily
Q#746 - >486382__F6AHOR	206837	43	79	3.00012e-16	69.9215	cl00101	KU superfamily
Q#747 - >486382__F6AHOR	206837	1	21	1.05182e-06	44.1131	cl00101	KU superfamily
Q#753 - >489552__F6AHOR	206837	12	65	1.5906e-21	83.7887	cl00101	KU superfamily
Q#759 - >490217__F6AHOR	206837	1	33	5.22572e-09	49.1207	cl00101	KU superfamily
Q#770 - >494438__F6AHOR	206837	32	76	1.18211e-20	82.2479	cl00101	KU superfamily
Q#771 - >494438__F6AHOR	206837	1	21	2.16253e-06	42.9575	cl00101	KU superfamily
Q#776 - >497749__F6AHOR	206837	55	92	1.00043e-14	65.2991	cl00101	KU superfamily
Q#783 - >500153__F6AHOR	206837	40	69	1.1311e-06	43.3427	cl00101	KU superfamily
Q#795 - >505741__F6AHOR	206837	5	58	1.55494e-15	67.2251	cl00101	KU superfamily
Q#811 - >516085__F6AHOR	206837	50	83	9.20762e-14	62.9879	cl00101	KU superfamily
Q#819 - >519374__F6AHOR	206837	1	27	8.23593e-09	50.2763	cl00101	KU superfamily
Q#823 - >524899__F6AHOR	206837	35	78	3.89509e-08	47.1947	cl00101	KU superfamily
Q#823 - >524899__F6AHOR	206837	80	111	2.10784e-06	42.2511	cl00101	KU superfamily
Q#825 - >524899__F6AHOR	206837	110	131	0.00953466	32.2706	cl00101	KU superfamily
Q#830 - >527095__F6AHOR	206837	77	106	1.9529e-08	47.9651	cl00101	KU superfamily
Q#831 - >527095__F6AHOR	206837	58	75	9.31871e-05	38.0486	cl00101	KU superfamily
Q#835 - >527127__F6AHOR	206837	43	91	5.12645e-21	82.2479	cl00101	KU superfamily
Q#841 - >527367__F6AHOR	206837	77	114	1.15602e-13	61.8323	cl00101	KU superfamily
Q#848 - >527587__F6AHOR	206837	19	71	5.91195e-07	43.4067	cl00101	KU superfamily
Q#855 - >532824__F6AHOR	206837	12	65	4.93064e-20	80.7071	cl00101	KU superfamily
Q#861 - >533064__F6AHOR	206837	10	35	1.18379e-05	40.7449	cl00101	KU superfamily

Table S4. Conserved domain hits for transcripts from *H. digitata*.

Query	PSSM-ID	From	To	E-Value	Bitscore	Accession	Short name
Q#1 - >11030__F6AHOR3	206837	67	120	2.37895e-16	69.5363	cl00101	KU superfamily
Q#1 - >11030__F6AHOR3	206837	21	65	3.13787e-13	61.0619	cl00101	KU superfamily
Q#8 - >15798__F6AHOR3	206837	14	66	9.22877e-18	73.3883	cl00101	KU superfamily
Q#14 - >17716__F6AHOR3	206837	5	42	5.63908e-13	60.2915	cl00101	KU superfamily
Q#15 - >17716__F6AHOR3	206837	62	115	5.56268e-20	79.1663	cl00101	KU superfamily
Q#19 - >19614__F6AHOR3	206837	24	76	6.02486e-17	71.0771	cl00101	KU superfamily
Q#19 - >19614__F6AHOR3	206837	82	135	7.14784e-17	70.6919	cl00101	KU superfamily
Q#26 - >21912__F6AHOR3	206834	19	59	9.47291e-07	42.2015	cl00097	KAZAL_FS superfamily
Q#26 - >21912__F6AHOR3	206837	73	116	7.07491e-06	40.2611	cl00101	KU superfamily
Q#32 - >22787__F6AHOR3	206837	55	107	4.71983e-19	76.8551	cl00101	KU superfamily
Q#38 - >37382__F6AHOR3	206837	8	57	2.33086e-12	58.3655	cl00101	KU superfamily
Q#45 - >47053__F6AHOR3	206837	39	91	1.6203e-16	69.9215	cl00101	KU superfamily
Q#45 - >47053__F6AHOR3	206837	1	37	0.000113337	37.2782	cl00101	KU superfamily
Q#51 - >47453__F6AHOR3	206837	53	106	2.89042e-20	79.5515	cl00101	KU superfamily
Q#56 - >52055__F6AHOR3	206837	1	47	9.73219e-20	78.3959	cl00101	KU superfamily
Q#62 - >58283__F6AHOR3	210239	1	35	8.67185e-05	37.0492	cl15839	ShK superfamily
Q#67 - >60139__F6AHOR3	206837	54	107	9.49689e-18	73.0031	cl00101	KU superfamily
Q#75 - >62342__F6AHOR3	109750	64	105	2.60806e-05	38.9921	cl02937	Toxin_4 superfamily
Q#81 - >71393__F6AHOR3	109750	64	106	0.000105549	37.8365	cl02937	Toxin_4 superfamily
Q#85 - >73916__F6AHOR3	206837	103	152	6.49015e-18	73.7735	cl00101	KU superfamily
Q#91 - >73949__F6AHOR3	206837	111	130	0.000177065	36.893	cl00101	KU superfamily
Q#93 - >73949__F6AHOR3	206837	130	161	1.3407e-05	39.9745	cl00101	KU superfamily
Q#97 - >77215__F6AHOR3	206837	39	89	4.56801e-20	79.1663	cl00101	KU superfamily
Q#103 - >79253__F6AHOR3	206837	58	111	4.52051e-18	74.1587	cl00101	KU superfamily
Q#109 - >80503__F6AHOR3	206837	108	137	1.43138e-08	47.9651	cl00101	KU superfamily
Q#111 - >80503__F6AHOR3	206837	85	105	6.31744e-05	38.4338	cl00101	KU superfamily
Q#116 - >81965__F6AHOR3	206837	20	72	1.60694e-18	75.3143	cl00101	KU superfamily
Q#128 - >84436__F6AHOR3	206837	1	48	1.48321e-16	69.5363	cl00101	KU superfamily
Q#135 - >84769__F6AHOR3	206837	71	114	2.97193e-14	63.3731	cl00101	KU superfamily
Q#140 - >86011__F6AHOR3	206837	110	123	0.00487269	32.6558	cl00101	KU superfamily
Q#141 - >86011__F6AHOR3	206837	81	111	1.63267e-06	42.1871	cl00101	KU superfamily
Q#145 - >87981__F6AHOR3	206837	48	98	6.00278e-23	86.8702	cl00101	KU superfamily
Q#153 - >97322__F6AHOR3	206837	3	55	2.08671e-17	72.2327	cl00101	KU superfamily
Q#153 - >97322__F6AHOR3	206837	61	114	2.50843e-17	72.2327	cl00101	KU superfamily
Q#157 - >99212__F6AHOR3	206837	37	89	7.27845e-19	76.0847	cl00101	KU superfamily
Q#164 - >99275__F6AHOR3	206837	71	102	7.30272e-11	54.1283	cl00101	KU superfamily
Q#170 - >99610__F6AHOR3	206837	58	111	4.94896e-19	76.4699	cl00101	KU superfamily
Q#177 - >105411__F6AHOR	206834	20	60	1.07629e-06	42.2015	cl00097	KAZAL_FS superfamily
Q#177 - >105411__F6AHOR	206837	74	117	6.74068e-06	40.2611	cl00101	KU superfamily
Q#183 - >107262__F6AHOR	206837	29	79	1.03265e-18	75.6995	cl00101	KU superfamily
Q#188 - >111656__F6AHOR	206837	1	42	6.72471e-10	51.4319	cl00101	KU superfamily
Q#195 - >114672__F6AHOR	206837	4	36	5.61506e-12	57.5951	cl00101	KU superfamily
Q#200 - >115529__F6AHOR	206837	125	142	0.000601597	35.3175	cl00101	KU superfamily
Q#201 - >115529__F6AHOR	206837	92	121	7.23315e-06	40.6463	cl00101	KU superfamily
Q#205 - >117052__F6AHOR	207622	83	146	8.7964e-06	41.3419	cl02512	NTR_like superfamily
Q#207 - >117052__F6AHOR	206837	1	36	7.43594e-13	59.9063	cl00101	KU superfamily
Q#213 - >119172__F6AHOR	206837	58	111	4.78691e-19	76.4699	cl00101	KU superfamily
Q#218 - >120641__F6AHOR	206837	79	124	2.27243e-18	74.9291	cl00101	KU superfamily
Q#225 - >121730__F6AHOR	206837	84	127	2.59837e-13	61.0619	cl00101	KU superfamily
Q#231 - >124434__F6AHOR	206837	66	104	5.83167e-11	54.5135	cl00101	KU superfamily

Table S4. *Cont.*

Q#237 - >131471__F6AHOR	206837	49	100	7.02588e-21	81.8627	cl00101	KU superfamily
Q#242 - >133440__F6AHOR	206837	83	133	3.31127e-16	69.1511	cl00101	KU superfamily
Q#247 - >134955__F6AHOR	206837	58	111	5.90731e-18	73.7735	cl00101	KU superfamily
Q#253 - >135085__F6AHOR	206837	39	89	4.27222e-20	79.5515	cl00101	KU superfamily
Q#260 - >136518__F6AHOR	206834	1	36	2.36178e-07	44.5127	cl00097	KAZAL_FS superfamily
Q#261 - >136518__F6AHOR	206837	58	111	1.66725e-18	75.3143	cl00101	KU superfamily
Q#266 - >137751__F6AHOR	109750	35	76	0.000181238	36.6809	cl02937	Toxin_4 superfamily
Q#271 - >139581__F6AHOR	206834	57	97	9.22727e-07	42.9719	cl00097	KAZAL_FS superfamily
Q#271 - >139581__F6AHOR	206837	111	149	0.000132683	37.2782	cl00101	KU superfamily
Q#277 - >140265__F6AHOR	206837	12	62	6.47335e-20	79.1663	cl00101	KU superfamily
Q#283 - >141682__F6AHOR	206837	85	141	8.17835e-10	51.0467	cl00101	KU superfamily
Q#290 - >143363__F6AHOR	206837	18	70	2.99638e-19	76.4699	cl00101	KU superfamily
Q#296 - >146922__F6AHOR	206837	49	102	1.02135e-18	76.0847	cl00101	KU superfamily
Q#301 - >151351__F6AHOR	206837	2	46	4.48254e-13	61.8323	cl00101	KU superfamily
Q#307 - >155688__F6AHOR	206837	26	75	3.22433e-13	61.0619	cl00101	KU superfamily
Q#314 - >160976__F6AHOR	206837	3	46	3.69575e-17	71.8475	cl00101	KU superfamily
Q#321 - >161654__F6AHOR	206837	41	94	2.8541e-17	71.8475	cl00101	KU superfamily
Q#325 - >162371__F6AHOR	206834	49	89	3.0378e-06	41.4311	cl00097	KAZAL_FS superfamily
Q#325 - >162371__F6AHOR	206837	103	146	5.46051e-06	41.1301	cl00101	KU superfamily
Q#332 - >162479__F6AHOR	109750	42	83	5.26734e-05	37.4513	cl02937	Toxin_4 superfamily
Q#337 - >162713__F6AHOR	206837	1	43	6.81175e-12	57.2099	cl00101	KU superfamily
Q#337 - >162713__F6AHOR	206834	71	111	5.47564e-08	46.1334	cl00097	KAZAL_FS superfamily
Q#343 - >166943__F6AHOR	206837	42	94	2.84475e-16	69.1511	cl00101	KU superfamily
Q#343 - >166943__F6AHOR	206837	1	40	9.44599e-05	37.6634	cl00101	KU superfamily
Q#351 - >169271__F6AHOR	206837	44	97	2.10401e-16	69.9215	cl00101	KU superfamily
Q#351 - >169271__F6AHOR	206834	1	40	2.8903e-07	44.1426	cl00097	KAZAL_FS superfamily
Q#351 - >169271__F6AHOR	206834	125	157	1.78342e-07	44.9778	cl00097	KAZAL_FS superfamily
Q#355 - >173288__F6AHOR	206837	69	122	2.95369e-21	82.6331	cl00101	KU superfamily
Q#361 - >175012__F6AHOR	206837	15	67	8.1074e-17	70.3067	cl00101	KU superfamily
Q#368 - >175091__F6AHOR	109750	34	75	0.000263991	36.2957	cl02937	Toxin_4 superfamily
Q#378 - >180148__F6AHOR	206837	83	136	1.77432e-14	64.1435	cl00101	KU superfamily
Q#381 - >186037__F6AHOR	206837	69	118	3.93996e-17	71.0771	cl00101	KU superfamily
Q#385 - >186240__F6AHOR	206837	41	94	6.26184e-18	73.7735	cl00101	KU superfamily
Q#391 - >187106__F6AHOR	206837	90	139	1.61877e-15	67.2251	cl00101	KU superfamily
Q#399 - >190791__F6AHOR	206837	69	118	5.06874e-17	71.0771	cl00101	KU superfamily
Q#404 - >195376__F6AHOR	206837	2	51	7.45294e-14	62.2175	cl00101	KU superfamily
Q#416 - >197752__F6AHOR	206837	49	98	7.28501e-15	65.2991	cl00101	KU superfamily
Q#421 - >199000__F6AHOR	109750	65	96	0.00227039	33.9845	cl02937	Toxin_4 superfamily
Q#427 - >200465__F6AHOR	206837	54	107	2.33429e-17	72.2327	cl00101	KU superfamily
Q#435 - >203242__F6AHOR	206837	41	94	4.73167e-18	74.5439	cl00101	KU superfamily
Q#440 - >203423__F6AHOR	206837	81	133	6.89083e-15	65.2991	cl00101	KU superfamily
Q#440 - >203423__F6AHOR	206837	36	79	4.94137e-06	41.0315	cl00101	KU superfamily
Q#445 - >209785__F6AHOR	206837	1	35	1.52168e-11	56.0543	cl00101	KU superfamily
Q#445 - >209785__F6AHOR	206834	68	102	7.62248e-06	40.2755	cl00097	KAZAL_FS superfamily
Q#446 - >209785__F6AHOR	206868	110	153	1.66389e-05	39.2747	cl00156	WAP superfamily
Q#453 - >212115__F6AHOR	109750	64	105	4.30544e-06	40.9181	cl02937	Toxin_4 superfamily
Q#464 - >217440__F6AHOR	206837	49	102	1.80421e-17	72.2327	cl00101	KU superfamily
Q#470 - >221150__F6AHOR	206837	25	78	1.24784e-21	83.0183	cl00101	KU superfamily
Q#477 - >223951__F6AHOR	206837	114	156	5.51423e-18	73.7735	cl00101	KU superfamily
Q#481 - >226390__F6AHOR	206837	17	70	2.78045e-22	84.9442	cl00101	KU superfamily
Q#487 - >230140__F6AHOR	206837	142	163	6.47717e-06	41.0315	cl00101	KU superfamily

Table S4. *Cont.*

Q#489 - >230140__F6AHOR	206837	112	141	1.02787e-05	40.3597	cl00101	KU superfamily
Q#494 - >235690__F6AHOR	109750	42	83	3.16809e-05	38.6069	cl02937	Toxin_4 superfamily
Q#504 - >238411__F6AHOR	206837	118	175	3.03335e-12	58.3655	cl00101	KU superfamily
Q#505 - >238669__F6AHOR	206837	5	33	2.90435e-06	41.5153	cl00101	KU superfamily
Q#507 - >238669__F6AHOR	206837	31	53	5.08473e-05	38.3991	cl00101	KU superfamily
Q#512 - >238782__F6AHOR	206837	56	109	8.03087e-19	76.0847	cl00101	KU superfamily
Q#517 - >244026__F6AHOR	206834	113	147	3.51401e-07	44.1275	cl00097	KAZAL_FS superfamily
Q#519 - >244026__F6AHOR	206837	26	79	1.16295e-16	70.3067	cl00101	KU superfamily
Q#524 - >249757__F6AHOR	206837	104	146	1.80106e-18	75.3143	cl00101	KU superfamily
Q#536 - >250910__F6AHOR	206837	31	84	6.37115e-17	71.0771	cl00101	KU superfamily
Q#542 - >250961__F6AHOR	206837	69	121	1.04399e-18	75.6995	cl00101	KU superfamily
Q#542 - >250961__F6AHOR	206837	127	152	0.000593445	35.3175	cl00101	KU superfamily
Q#547 - >256926__F6AHOR	206837	58	111	8.22329e-18	73.3883	cl00101	KU superfamily
Q#554 - >257732__F6AHOR	206837	101	158	1.20428e-12	59.1359	cl00101	KU superfamily
Q#560 - >258498__F6AHOR	206837	1	39	7.46997e-11	54.1283	cl00101	KU superfamily
Q#565 - >259588__F6AHOR	206837	24	76	1.57646e-16	69.9215	cl00101	KU superfamily
Q#565 - >259588__F6AHOR	206837	82	135	1.87548e-16	69.5363	cl00101	KU superfamily
Q#571 - >260799__F6AHOR	206837	51	102	7.66365e-16	68.3807	cl00101	KU superfamily
Q#571 - >260799__F6AHOR	206837	1	41	6.51182e-12	57.2099	cl00101	KU superfamily
Q#577 - >263762__F6AHOR	206837	42	94	3.05226e-18	74.5439	cl00101	KU superfamily
Q#583 - >264832__F6AHOR	206837	54	107	6.7575e-18	73.3883	cl00101	KU superfamily
Q#591 - >265534__F6AHOR	206834	50	90	3.35103e-08	46.5186	cl00097	KAZAL_FS superfamily
Q#591 - >265534__F6AHOR	206837	1	22	2.51255e-05	38.8189	cl00101	KU superfamily
Q#597 - >269593__F6AHOR	206837	1	45	4.4545e-15	65.6843	cl00101	KU superfamily
Q#602 - >271395__F6AHOR	206837	55	93	1.60981e-10	52.9727	cl00101	KU superfamily
Q#603 - >271395__F6AHOR	206837	88	107	0.00594247	32.2706	cl00101	KU superfamily
Q#608 - >271511__F6AHOR	206837	23	76	2.75696e-16	69.5363	cl00101	KU superfamily
Q#608 - >271511__F6AHOR	206834	104	130	5.3583e-05	38.0442	cl00097	KAZAL_FS superfamily
Q#614 - >276325__F6AHOR	206837	57	110	2.38738e-17	71.8475	cl00101	KU superfamily
Q#619 - >280586__F6AHOR	206837	94	143	1.62218e-08	47.9651	cl00101	KU superfamily
Q#626 - >281064__F6AHOR	206837	75	104	3.54631e-09	49.5059	cl00101	KU superfamily
Q#631 - >284257__F6AHOR	206837	42	95	1.20464e-18	75.6995	cl00101	KU superfamily
Q#638 - >285673__F6AHOR	206837	81	124	1.16497e-08	47.9651	cl00101	KU superfamily
Q#643 - >285843__F6AHOR	206837	23	76	1.77535e-17	72.6179	cl00101	KU superfamily
Q#643 - >285843__F6AHOR	206834	104	144	2.47448e-09	49.9854	cl00097	KAZAL_FS superfamily
Q#650 - >289615__F6AHOR	206837	98	114	2.25363e-05	38.4338	cl00101	KU superfamily
Q#651 - >289615__F6AHOR	206837	71	97	1.88375e-05	38.8189	cl00101	KU superfamily
Q#655 - >292620__F6AHOR	206837	59	109	3.31015e-16	69.1511	cl00101	KU superfamily
Q#661 - >292829__F6AHOR	206837	22	74	2.30299e-17	72.2327	cl00101	KU superfamily
Q#661 - >292829__F6AHOR	206837	80	123	1.25074e-07	45.2687	cl00101	KU superfamily
Q#669 - >293184__F6AHOR	109750	64	105	3.3565e-05	38.6069	cl02937	Toxin_4 superfamily
Q#674 - >298272__F6AHOR	109750	34	75	4.25402e-05	38.2217	cl02937	Toxin_4 superfamily
Q#681 - >300778__F6AHOR	206837	95	129	1.37772e-08	47.9651	cl00101	KU superfamily
Q#687 - >309346__F6AHOR	206837	122	144	1.27691e-06	42.5723	cl00101	KU superfamily
Q#692 - >318170__F6AHOR	206837	61	112	1.92079e-19	77.6255	cl00101	KU superfamily
Q#699 - >318836__F6AHOR	206837	102	155	2.28781e-14	64.1435	cl00101	KU superfamily
Q#699 - >318836__F6AHOR	206834	3	43	1.09845e-06	42.5867	cl00097	KAZAL_FS superfamily
Q#699 - >318836__F6AHOR	206837	57	100	3.56805e-06	41.5153	cl00101	KU superfamily
Q#704 - >326281__F6AHOR	206837	40	93	4.90486e-17	71.4623	cl00101	KU superfamily
Q#704 - >326281__F6AHOR	206834	1	36	3.76773e-08	46.5186	cl00097	KAZAL_FS superfamily
Q#709 - >327156__F6AHOR	206837	2	46	1.14494e-14	64.5287	cl00101	KU superfamily

Table S4. *Cont.*

Q#715 - >329908__F6AHOR	109750	62	103	7.46574e-05	37.8365	cl02937	Toxin_4 superfamily
Q#721 - >331879__F6AHOR	206868	132	152	0.00398108	32.8024	cl00156	WAP superfamily
Q#723 - >331879__F6AHOR	206837	1	34	3.25886e-11	55.2839	cl00101	KU superfamily
Q#723 - >331879__F6AHOR	206834	62	102	7.61108e-10	51.141	cl00097	KAZAL_FS superfamily
Q#729 - >337351__F6AHOR	206837	86	118	3.02573e-10	52.5875	cl00101	KU superfamily
Q#734 - >339734__F6AHOR	206837	110	156	4.0693e-18	74.1587	cl00101	KU superfamily
Q#740 - >344132__F6AHOR	206837	21	73	1.20737e-17	73.3883	cl00101	KU superfamily
Q#746 - >345772__F6AHOR	206837	117	147	1.55806e-08	47.9651	cl00101	KU superfamily
Q#747 - >345772__F6AHOR	206837	98	116	0.00574358	33.041	cl00101	KU superfamily
Q#751 - >346986__F6AHOR	206837	49	89	4.06482e-12	57.9803	cl00101	KU superfamily
Q#751 - >346986__F6AHOR	206837	4	47	1.44711e-07	45.2687	cl00101	KU superfamily
Q#758 - >352982__F6AHOR	206837	49	101	1.75979e-21	83.0183	cl00101	KU superfamily
Q#762 - >352982__F6AHOR	150200	71	98	0.00156131	34.1869	cl09685	VMA21 superfamily
Q#763 - >353606__F6AHOR	206837	80	120	6.972e-07	42.9575	cl00101	KU superfamily
Q#764 - >353606__F6AHOR	206834	26	56	3.46658e-06	41.1663	cl00097	KAZAL_FS superfamily
Q#771 - >353983__F6AHOR	109750	33	74	5.62871e-05	37.8365	cl02937	Toxin_4 superfamily
Q#775 - >357651__F6AHOR	206837	56	109	1.71418e-18	74.9291	cl00101	KU superfamily
Q#782 - >358541__F6AHOR	206837	61	100	3.33309e-11	55.2839	cl00101	KU superfamily
Q#793 - >361798__F6AHOR	206837	45	98	1.03794e-17	73.0031	cl00101	KU superfamily
Q#793 - >361798__F6AHOR	206837	2	39	7.49209e-12	56.8247	cl00101	KU superfamily
Q#801 - >366690__F6AHOR	109750	64	105	2.122e-05	39.3773	cl02937	Toxin_4 superfamily
Q#805 - >368814__F6AHOR	206837	21	74	7.56244e-17	70.6919	cl00101	KU superfamily
Q#805 - >368814__F6AHOR	206863	95	123	2.56183e-06	42.0178	cl00150	TY superfamily
Q#812 - >373573__F6AHOR	206837	31	84	1.43349e-16	69.9215	cl00101	KU superfamily
Q#812 - >373573__F6AHOR	206834	112	143	2.01953e-07	44.5926	cl00097	KAZAL_FS superfamily
Q#818 - >376106__F6AHOR	206837	1	47	4.804e-20	79.1663	cl00101	KU superfamily
Q#825 - >377697__F6AHOR	206837	75	110	2.18039e-11	56.0543	cl00101	KU superfamily
Q#831 - >381474__F6AHOR	109750	44	85	2.78065e-05	38.6069	cl02937	Toxin_4 superfamily
Q#836 - >388614__F6AHOR	109750	66	107	4.89182e-05	38.2217	cl02937	Toxin_4 superfamily
Q#842 - >392330__F6AHOR	206837	134	162	4.08116e-09	49.1207	cl00101	KU superfamily
Q#848 - >394828__F6AHOR	206837	99	118	5.40659e-06	40.7449	cl00101	KU superfamily
Q#849 - >394828__F6AHOR	206837	113	137	1.14898e-05	39.9745	cl00101	KU superfamily
Q#855 - >396124__F6AHOR	206837	23	76	2.24533e-16	69.5363	cl00101	KU superfamily
Q#855 - >396124__F6AHOR	206834	104	128	0.000105153	37.2738	cl00097	KAZAL_FS superfamily
Q#859 - >398513__F6AHOR	206837	25	78	1.49928e-21	83.0183	cl00101	KU superfamily
Q#867 - >401808__F6AHOR	109750	64	85	0.000879574	35.5253	cl02937	Toxin_4 superfamily
Q#871 - >404905__F6AHOR	206837	60	112	8.35602e-17	71.0771	cl00101	KU superfamily
Q#878 - >406361__F6AHOR	109750	53	94	4.31536e-05	38.2217	cl02937	Toxin_4 superfamily
Q#883 - >408752__F6AHOR	206837	23	66	4.1575e-07	44.1131	cl00101	KU superfamily
Q#883 - >408752__F6AHOR	206837	68	97	1.69882e-05	39.5547	cl00101	KU superfamily
Q#885 - >408752__F6AHOR	206837	92	119	3.24854e-08	46.8095	cl00101	KU superfamily
Q#890 - >409393__F6AHOR	206837	41	91	1.41359e-16	70.3067	cl00101	KU superfamily
Q#895 - >413841__F6AHOR	206837	93	125	9.42803e-09	48.7355	cl00101	KU superfamily
Q#901 - >413913__F6AHOR	206837	2	46	2.12942e-11	55.6691	cl00101	KU superfamily
Q#907 - >418618__F6AHOR	206834	49	89	2.8721e-08	46.5186	cl00097	KAZAL_FS superfamily
Q#907 - >418618__F6AHOR	206837	1	21	0.000233204	36.5078	cl00101	KU superfamily
Q#907 - >418618__F6AHOR	206868	96	139	0.00893084	31.9559	cl00156	WAP superfamily
Q#913 - >419072__F6AHOR	206837	62	84	8.80356e-06	40.6463	cl00101	KU superfamily
Q#914 - >419072__F6AHOR	206837	86	112	5.85842e-10	51.8171	cl00101	KU superfamily
Q#920 - >419247__F6AHOR	206837	1	47	4.40458e-19	76.8551	cl00101	KU superfamily
Q#925 - >421339__F6AHOR	206837	23	76	6.59404e-17	71.0771	cl00101	KU superfamily

Table S4. *Cont.*

Q#925 - >421339__F6AHOR	206834	104	136	1.27689e-06	42.6666	cl00097	KAZAL_FS superfamily
Q#932 - >421660__F6AHOR	206837	80	133	3.87898e-17	71.4623	cl00101	KU superfamily
Q#932 - >421660__F6AHOR	206837	28	74	1.73782e-14	64.1435	cl00101	KU superfamily
Q#937 - >422304__F6AHOR	206837	90	133	7.35693e-08	46.0391	cl00101	KU superfamily
Q#939 - >422304__F6AHOR	206834	32	66	1.22069e-06	42.6666	cl00097	KAZAL_FS superfamily
Q#939 - >422304__F6AHOR	206837	134	161	1.5989e-05	39.5547	cl00101	KU superfamily
Q#944 - >424241__F6AHOR	206837	58	111	6.35668e-18	73.7735	cl00101	KU superfamily
Q#949 - >426449__F6AHOR	206837	5	57	8.89994e-19	75.6995	cl00101	KU superfamily
Q#950 - >426449__F6AHOR	206837	77	106	1.57167e-09	50.2763	cl00101	KU superfamily
Q#957 - >428248__F6AHOR	109750	31	72	0.000132678	36.6809	cl02937	Toxin_4 superfamily
Q#961 - >432797__F6AHOR	206837	31	84	1.81981e-21	83.0183	cl00101	KU superfamily
Q#967 - >435285__F6AHOR	206837	55	91	1.37061e-09	50.6615	cl00101	KU superfamily
Q#973 - >436730__F6AHOR	206837	92	145	1.84033e-21	83.0183	cl00101	KU superfamily
Q#981 - >437310__F6AHOR	206837	35	87	1.43356e-16	69.9215	cl00101	KU superfamily
Q#986 - >437369__F6AHOR	206837	55	106	9.00227e-20	78.7811	cl00101	KU superfamily
Q#991 - >438161__F6AHOR	206837	96	139	9.48882e-11	53.7431	cl00101	KU superfamily
Q#997 - >439437__F6AHOR	206837	109	151	1.03117e-14	64.9139	cl00101	KU superfamily
Q#1005 - >444641__F6AHOR	206837	78	115	1.05223e-09	51.0467	cl00101	KU superfamily
Q#1009 - >444778__F6AHOR	206837	61	113	4.69655e-23	87.2554	cl00101	KU superfamily
Q#1017 - >446516__F6AHOR	109750	52	93	1.63268e-05	39.3773	cl02937	Toxin_4 superfamily
Q#1023 - >448262__F6AHOR	206837	25	77	1.18255e-19	78.0107	cl00101	KU superfamily
Q#1028 - >448805__F6AHOR	206837	55	107	3.06189e-18	74.9291	cl00101	KU superfamily
Q#1034 - >449621__F6AHOR	206837	36	85	1.32744e-18	76.0847	cl00101	KU superfamily
Q#1045 - >454341__F6AHOR	206837	37	89	8.987e-20	78.7811	cl00101	KU superfamily
Q#1051 - >458453__F6AHOR	206837	71	124	1.44789e-21	83.4035	cl00101	KU superfamily
Q#1058 - >459665__F6AHOR	206837	113	155	5.14627e-15	65.6843	cl00101	KU superfamily
Q#1065 - >465507__F6AHOR	206837	58	107	1.57754e-17	72.6179	cl00101	KU superfamily
Q#1071 - >465535__F6AHOR	109750	64	88	0.000144277	37.8365	cl02937	Toxin_4 superfamily
Q#1075 - >468848__F6AHOR	206837	103	116	0.000561972	35.7027	cl00101	KU superfamily
Q#1076 - >468848__F6AHOR	206837	29	72	1.1097e-08	48.3503	cl00101	KU superfamily
Q#1077 - >468848__F6AHOR	206837	78	101	0.000162471	37.2435	cl00101	KU superfamily
Q#1081 - >471349__F6AHOR	206837	85	122	1.52082e-07	44.8835	cl00101	KU superfamily
Q#1083 - >471349__F6AHOR	206837	1	40	1.4487e-14	64.5287	cl00101	KU superfamily
Q#1087 - >474922__F6AHOR	206837	81	115	1.05842e-08	48.3503	cl00101	KU superfamily
Q#1094 - >475566__F6AHOR	206837	85	128	1.45007e-13	61.8323	cl00101	KU superfamily
Q#1100 - >481884__F6AHOR	206837	35	78	5.25097e-07	43.7279	cl00101	KU superfamily
Q#1101 - >481884__F6AHOR	206837	96	131	9.86218e-12	56.8247	cl00101	KU superfamily
Q#1105 - >484973__F6AHOR	206837	19	65	1.01193e-15	67.9955	cl00101	KU superfamily
Q#1105 - >484973__F6AHOR	206837	71	110	1.84424e-11	56.0543	cl00101	KU superfamily
Q#1112 - >485015__F6AHOR	206837	58	111	1.83971e-18	74.9291	cl00101	KU superfamily
Q#1117 - >488941__F6AHOR	206837	60	113	5.20435e-17	71.0771	cl00101	KU superfamily
Q#1124 - >491009__F6AHOR	206837	80	103	0.000383141	35.6387	cl00101	KU superfamily
Q#1125 - >491009__F6AHOR	206837	49	85	3.43877e-12	57.9803	cl00101	KU superfamily
Q#1130 - >491360__F6AHOR	206837	74	102	6.68435e-10	52.2023	cl00101	KU superfamily
Q#1131 - >491360__F6AHOR	206837	11	52	1.87209e-14	64.9139	cl00101	KU superfamily
Q#1137 - >492790__F6AHOR	206837	3	55	2.66982e-16	69.1511	cl00101	KU superfamily
Q#1137 - >492790__F6AHOR	206837	61	104	2.0154e-12	58.3655	cl00101	KU superfamily
Q#1142 - >503084__F6AHOR	206837	37	88	2.12131e-17	72.2327	cl00101	KU superfamily
Q#1149 - >504175__F6AHOR	109750	54	95	9.33378e-05	37.8365	cl02937	Toxin_4 superfamily
Q#1155 - >506530__F6AHOR	206837	57	108	5.62632e-16	68.7659	cl00101	KU superfamily
Q#1161 - >506891__F6AHOR	109750	64	95	0.00263492	33.5993	cl02937	Toxin_4 superfamily

Table S4. *Cont.*

Q#1167 - >512494__F6AHOR	206837	100	151	5.52957e-09	49.1207	cl00101	KU superfamily
Q#1172 - >517529__F6AHOR	206837	58	108	4.33615e-14	62.9879	cl00101	KU superfamily
Q#1177 - >521760__F6AHOR	206837	1	45	2.70616e-16	69.9215	cl00101	KU superfamily
Q#1183 - >531175__F6AHOR	206837	38	57	2.78936e-06	42.6709	cl00101	KU superfamily
Q#1185 - >531175__F6AHOR	206837	4	34	2.12052e-08	48.3503	cl00101	KU superfamily
Q#1191 - >531617__F6AHOR	206837	101	134	7.60457e-12	56.8247	cl00101	KU superfamily
Q#1197 - >534494__F6AHOR	109750	64	88	7.18292e-05	38.6069	cl02937	Toxin_4 superfamily
Q#1209 - >543217__F6AHOR	206837	9	53	2.06407e-11	56.0543	cl00101	KU superfamily
Q#1213 - >543244__F6AHOR	206837	1	34	1.95167e-10	52.9727	cl00101	KU superfamily
Q#1213 - >543244__F6AHOR	206834	62	102	3.55734e-09	49.215	cl00097	KAZAL_FS superfamily
Q#1220 - >544895__F6AHOR	109750	53	84	0.00213969	33.9845	cl02937	Toxin_4 superfamily
Q#1225 - >544997__F6AHOR	206837	47	90	2.70615e-16	70.6919	cl00101	KU superfamily
Q#1231 - >545861__F6AHOR	206837	1	54	1.63721e-16	69.9215	cl00101	KU superfamily

© 2012 by the authors; licensee MDPI, Basel, Switzerland. This article is an open access article distributed under the terms and conditions of the Creative Commons Attribution license (http://creativecommons.org/licenses/by/3.0/).
